# Investigating the *Campylobacter* enteritis winter peak in Germany, 2018/2019

**DOI:** 10.1038/s41598-021-02423-8

**Published:** 2021-11-25

**Authors:** Bettina M. Rosner, Martyna Gassowski, Stefan Albrecht, Klaus Stark

**Affiliations:** 1 Department of Infectious Disease Epidemiology, Robert Koch Institute, Berlin, Germany; 2 Department of Epidemiology and Health Monitoring, Robert Koch Institute, Berlin, Germany

**Keywords:** Diseases, Gastrointestinal diseases, Gastroenteritis, Risk factors

## Abstract

Surveillance of notified *Campylobacter* enteritis in Germany revealed a recurrent annual increase of cases with disease onset several days after the Christmas and New Year holidays (“winter peak”). We suspected that handling and consumption of chicken meat during fondue and raclette grill meals on the holidays were associated with winter peak *Campylobacter* infections. The hypothesis was investigated in a case–control study with a case-case design where notified *Campylobacter* enteritis cases served as case-patients as well as control-patients, depending on their date of disease onset (case-patients: 25/12/2018 to 08/01/2019; control-patients: any other date between 30/11/2018 and 28/02/2019). The study was conducted as an online survey from 21/01/2019 to 18/03/2019. Adjusted odds ratios (aOR) were determined in single-variable logistic regression analyses adjusted for age group and sex. We analysed 182 data sets from case-patients and 260 from control-patients and found associations of *Campylobacter* infections after the holidays with meat fondue (aOR 2.2; 95% confidence interval (CI) 1.2–3.8) and raclette grill meals with meat (aOR 1.5; 95% CI 1.0–2.4) consumed on the holidays. The associations were stronger when chicken meat was served at these meals (fondue with chicken meat: aOR 2.7; 95% CI 1.4–5.5; raclette grill meal with chicken meat: aOR 2.3; 95% CI 1.3–4.1). The results confirmed our initial hypothesis. To prevent *Campylobacter* winter peak cases in the future, consumers should be made more aware of the risks of a *Campylobacter* infection when handling raw meat, in particular chicken, during fondue or raclette grill meals on the holidays.

## Introduction

*Campylobacter* enteritis is the most common notifiable bacterial gastrointestinal disease in Germany. In the time period from 2010 to 2019 about 60,000 to 75,000 cases were notified per year (median 69,000 cases)^1,2^. As there is substantial underreporting, the number of *Campylobacter* enteritis cases in the population is assumed to be higher by a factor of about 5 to 10^3,4^. In a large case–control and source attribution study conducted in the years 2011–2014 we could demonstrate that preparation and consumption of chicken meat is the most important risk factor for sporadic *Campylobacter* infections in Germany, and that chicken is the most relevant source^5^.

Most infections in Germany occur in the summer months^1^. When analysing our surveillance data, we were intrigued by a recurrent annual increase of notified *Campylobacter* infections that occurred in the winter (“winter peak”) and were not related to traveling. The winter peak appeared to be specific for *Campylobacter* enteritis; it was not observed for salmonellosis, another notifiable foodborne infectious disease. A more detailed analysis of the *Campylobacter* enteritis surveillance data revealed not one but two winter peaks; one peak occurred at the end of December and a second peak in early January. The dates of disease onset indicated that the increase of *Campylobacter* enteritis in the surveillance data at the end of December and early January most likely resulted from infections in the preceding days, and was not due to reporting delays over the Christmas and New Year holidays^2^. The incubation period for *Campylobacter* enteritis is about 2–5 days^6,7^. Therefore, possible days of exposure of winter peak cases included the Christmas holidays (December 24–26) for those with disease onset in late December, and New Year’s Eve (December 31) or New Year’s Day (January 1) for those with disease onset in early January.

We suspected that the winter peak cases had been exposed to certain *Campylobacter*-contaminated food items consumed on those holidays. A similar increase of *Campylobacter* enteritis in the winter was also observed in Switzerland^8^. In a case–control study conducted in 2012/2013, Bless et al. identified the consumption of meat fondue in the week before disease onset as an important risk factor for winter peak *Campylobacter* enteritis in Switzerland. The association of consumption of meat fondue and disease was strongest when chicken meat had been served at the fondues^8^. Besides Germany and Switzerland, the winter peak was also apparent in several other European countries^9,10^. The authors suspected that meat fondue or tabletop grill meals may be popular holiday meals in these countries as well^9^. In our case–control study, conducted 2011 to 2014, we also observed an increase in cases with disease onset after the Christmas and the New Year holidays. Unfortunately, the questionnaire for that study did not include specific questions regarding consumption of meat fondue or tabletop grill meals and, therefore, at the time, we could not analyse if these exposures were risk factors for *Campylobacter* enteritis in Germany.

The objectives of the present study were as follows: (1) to confirm, based on routine surveillance date, that notified *Campylobacter* enteritis cases increased after the holidays in the year we conducted the study, (2) to investigate the hypothesis that in Germany consumption of meals on the Christmas or New Year’s holidays, where meat is heated directly at the table (e.g. meat fondue, raclette grill meals, other tabletop grill meals), was associated with *Campylobacter* enteritis with disease onset after the holidays, (3) to analyse if the type of meat consumed at meat fondue, raclette grill or similar meals on the holidays had an effect on the presumed association, (4) to investigate if certain behavioural aspects at those types of meals, such as touching the raw meat with bare fingers, placing raw and cooked meat on the same plate, and serving food items that remain uncooked at these meals, were factors contributing to *Campylobacter* infections. Our goal was to provide evidence for recommendations to help prevent future *Campylobacter* infections during the holidays.

## Methods

### Trend analysis of reported *Campylobacter* enteritis cases with disease onset after Christmas and New Year’s holidays

We analysed data of *Campylobacter* enteritis cases that were notified through the routine surveillance system for notifiable infectious diseases in Germany (SurvNet@rki) in the time period 2013/2014–2019/2020. We focused on cases that were notified to the local health authorities with disease onset in January or February of the respective year, or December of the preceding year. We included only cases that had reported exposure in Germany in the days before disease onset; travel-related cases were excluded. Cases that were notified without a date of disease onset were also excluded from analysis.

### Definition of time periods

For the remainder of this article, the time period from 1 December to 28 (or 29) February will be referred to as “winter season”. “Christmas holidays” are defined as 24, 25, and 26 December; “New Year’s holidays” are defined as 31 December and 1 January.

### Study design

The study was conducted as an online survey among *Campylobacter* patients that had been notified to the local health authorities. Notified *Campylobacter* cases served as cases or controls in our case–control study, depending on their date of disease onset (see below). Parents of notified *Campylobacter* enteritis patients younger than 15 years of age were asked to fill in the questionnaire for or with their child. The wording of the questionnaire for parents and their children was adapted respectively.

### Recruitment of study participants

On 21 January 2019 the recruitment process was officially started. On that day, state health authorities of all 16 federal states were asked to forward study information to the local health authorities within their state. In Germany, case-patients with a notified *Campylobacter* infection are contacted by the local health authorities as part of their routine investigations according to the Protection against Infection Act (Infektionsschutzgesetz). When contacting case-patients, the local health authorities were asked to include a recruitment letter, which explained the purpose of the study, data protection measures, and provided a link and QR code to access the online questionnaire. All case-patients with *Campylobacter* infections notified to the local health authorities in Germany between 2 January and 28 February 2019 were eligible for participation in the study. Local public health authorities from 15 of the 16 federal states (exception: Rhineland Palatinate) participated in the recruitment process.

### Online survey

The online survey was activated on 21 January 2019 and was accessible until 18 March 2019. Data sets from before 23 January 2019 were excluded from analysis. We assumed that *Campylobacter* enteritis cases did not access the online survey before 23 January 2019, because study information had to be forwarded from the state to the local health authorities, and from the local health authorities to eligible *Campylobacter* enteritis case-patients first. Therefore, we considered it as not plausible that data sets from before 23 January 2019 had been created by *Campylobacter* enteritis case-patients and suspected that these data sets had been created by employees of state or local health authorities who tested the online questionnaire after we had sent the link to the online survey.

### Response rate

We compared the number of data sets (complete or incomplete) with plausible access dates from the online survey to the overall number of non-travel related *Campylobacter* enteritis cases that were notified to the local health authorities of the participating federal states in the time period from 2 January to 28 February 2019. We defined the response rate as the number of complete data sets (participants had looked at the questions up to the end of the questionnaire and at least answered some of them) divided by the number of non-travel related *Campylobacter* enteritis cases that were notified to the local health authorities of the participating federal states overall in the same time period. We assumed that the local health authorities forwarded the study information to every eligible case notified to them. We also compared the number of participants in our case–control study and the total number of notified cases that would have been eligible for participation in our case–control study according to federal state.

### Questionnaire

The questionnaire was programmed in VOXCO Acuity™ Version 5.5.1.211 (Voxco, Montreal, CA). Photograph illustrations were included where considered helpful for the participants (e.g., photographs of fondue vs. raclette grill vs. other tabletop grills). A questionnaire version that allowed answering the questions on mobile phones was also available (adaptive design).

The focus of the questionnaire was on food items consumed on the Christmas and New Year’s holidays, in particular handling and consumption of meat as part of a fondue or raclette grill meal. Consumption of several other food items that are known or possible risk factors for *Campylobacter* infections (consumption of ground meat, poultry meat, unpasteurised milk) in the 7 days before disease onset was queried as well. We also queried illness-related information (symptoms, duration of symptoms, health-seeking behaviour, treatment, hospitalisation) (Supplementary S1). Diarrhoea was defined as passing of unformed stool at least 3 times within 24 h; liquid stool was defined as unformed stool that was passed fewer than 3 times within 24 h. Fever was defined as a body temperature of more than 38.5 °C. The questionnaire also contained a few demographic questions (month and year of birth, sex, postal code of place of residency).

### Case–control study

Data sets were excluded from data analysis if the date of participation was not plausible, participation was incomplete (i.e. last page of the questionnaire had not been reached), data sets did not contain any data, the date of disease onset was either missing or out of range (before 30 November 2018 or after 28 February 2019), or travel abroad in the 7 days prior to disease onset was indicated. Study participants were categorised as cases if their date of disease onset reported on the questionnaire was in the time period from 25 December 2018 to 8 January 2019 (1 to 7 days after the Christmas or the New Year’s holidays). Study participants were defined as controls if their date of disease onset reported on the questionnaire was within the time periods from 30 November to 24 December 2018, or from 9 January to 28 February 2019.

For more detailed analyses of subgroups, we categorised “Christmas Eve cases” as cases with a date of disease onset in the time period from 25 December 2018 to 31 December 2018, and “New Year’s Eve cases” as cases with a date of disease onset in the time period from 1 January 2019 to 7 January 2019 because Christmas Eve and New Year’s Eve were reported as the most popular dates for fondue or raclette grill meals in our study.

### Statistical analyses

For statistical analyses we used Stata 15.1 (Stata Corporation LLC, College Station, TX; USA). We performed descriptive analysis by calculating proportions, medians and ranges, where appropriate. Logistic regression analyses based on single exposure variables were adjusted for age group (0–14 years, 15–59 years, 60 years and older) and sex (“single variable analyses”) to determine adjusted odds ratios (aOR) with 95% confidence intervals (CI). Due to small case numbers in various strata multivariable logistic regression analyses were not conducted. Statistical significance was assessed using Wald tests. *P*-values < 0.05 were considered as an indication of statistical significance.

Assuming that a certain proportion of control-patients with disease onset in the 7 days before Christmas who did not have a meat fondue or raclette grill meal on the holidays and did not report any other food consumption on the holidays (n = 10) would have eaten fondue or raclette grill meals if they had not been ill, we conducted a simple sensitivity analysis. For this analysis, we arithmetically increased the number of control-patients with meat fondue exposure by 2, and of control-patients with raclette grill meal exposure by 3 (20% and 30%, respectively), and re-calculated the adjusted odds ratio.

### Data protection

The study design was approved by the data protection officer of the Robert Koch Institute. Data protection measures included adherence to EU’s general data protection regulation. Participation in the online survey was voluntary. In the online questionnaire, participants were given the opportunity to skip questions and to indicate that they did not want to answer demographic questions. Month and year of birth was not queried for participants who reported their age group as 80 years or older. Collected data were recorded anonymously.

### Ethics approval

Ethics approval was granted from the ethics committee of the Charité University Medicine, Berlin, Germany (Number EA2/217/18; 31 October 2018). The study was conducted in accordance with relevant guidelines and regulations.

### Consent to participate

Participation in the study was voluntary. Informed consent was obtained from all subjects who participated in the study or a parent or legal guardian if the study participant was a minor.

## Results

### Trend analysis of reported *Campylobacter* enteritis cases

**Figure 1:**
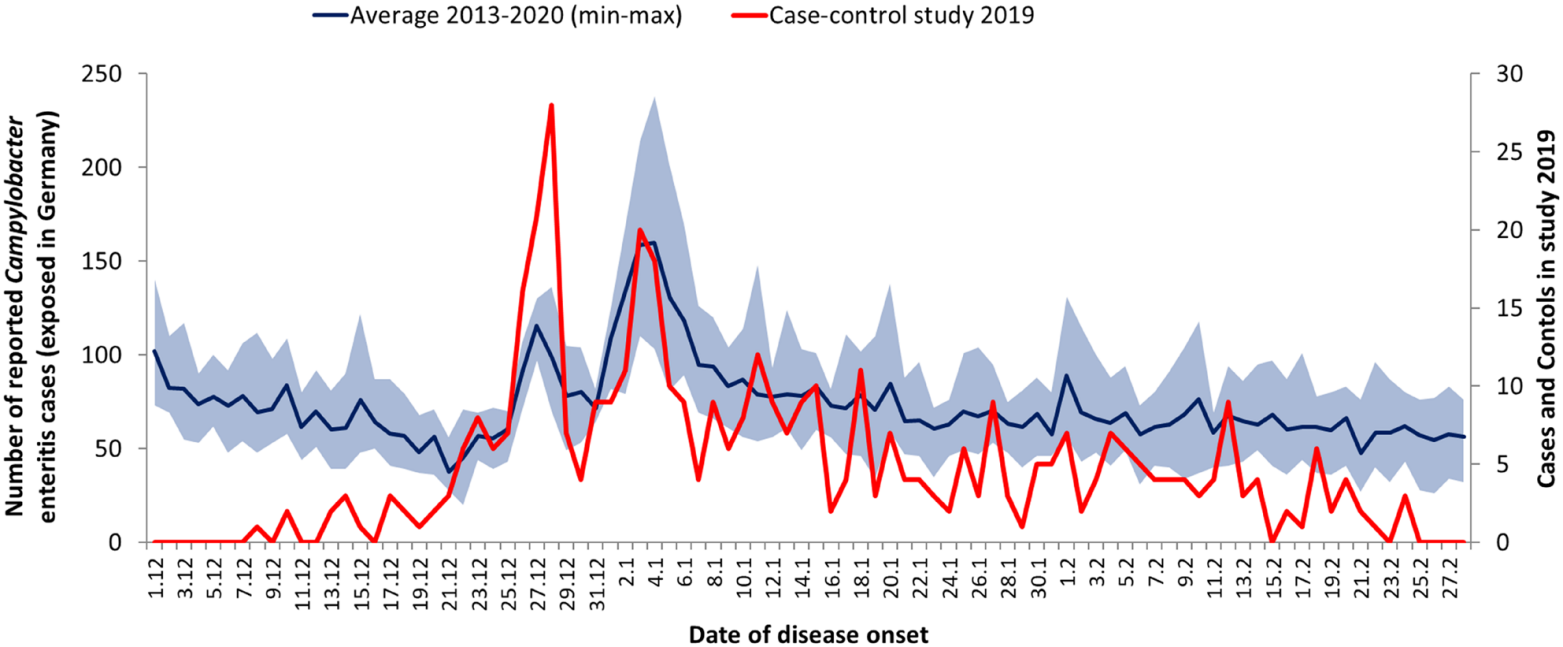
Average number of non-travel related *Campylobacter* enteritis cases notified to the RKI according to the Protection against Infection Act (Infektionsschutzgesetz) (blue line; minimum and maximum shown in the light blue area) by date of disease onset in the time period 2013/2014–2019/2020. The red line shows the date of disease onset of participants of the case–control study (cases with date of disease onset from 25 December 2018 to 8 January 2019; controls with dates of disease onset earlier or later).

We analysed notified, non-travel related *Campylobacter* enteritis cases in the winter seasons of the years 2013/14 to 2019/20. A sharp increase of notified *Campylobacter* enteritis cases with disease onset after the Christmas holidays (27 or 28 December; 70–136 cases per day) and after the New Year’s holidays (3 to 5 January; 81–238 cases per day) could be observed each year. The most commonly reported dates of disease onset were 27 and 28 December with a median of 104 cases per day for the time period 2013–2020, and 3 and 4 January with a median of 137 cases per day (Fig. 1). The increase of cases after New Year’s holidays was higher than the increase after the Christmas holidays, except in the year 2018/19, when we conducted our study (Fig. 1). The total number of notified autochthonous cases with disease onset between 1 December and 28 February decreased from 7946 in the winter season 2013/14 to 5412 in 2018/19. Correspondingly, the number of cases with disease onset after the holidays decreased from about 1900 in the years 2013/14 (1864 cases), 2014/15 (1922), and 2015/16 (1859) to 1208 in 2018/19, the year we conducted our study. The average number of cases with minimum and maximum values are depicted in Fig. 1. The proportion of cases with disease onset between 25 December and 8 January among all cases with disease onset in the winter season remained relatively stable, with an average of 24% (range 22–25%). In the winter season 2019/20, the decreasing trend in the number of cases with disease onset after the holidays did not continue and case numbers with disease onset between 25 December and 8 January were slightly higher again (n = 1305).

### Study participants

**Figure 2:**
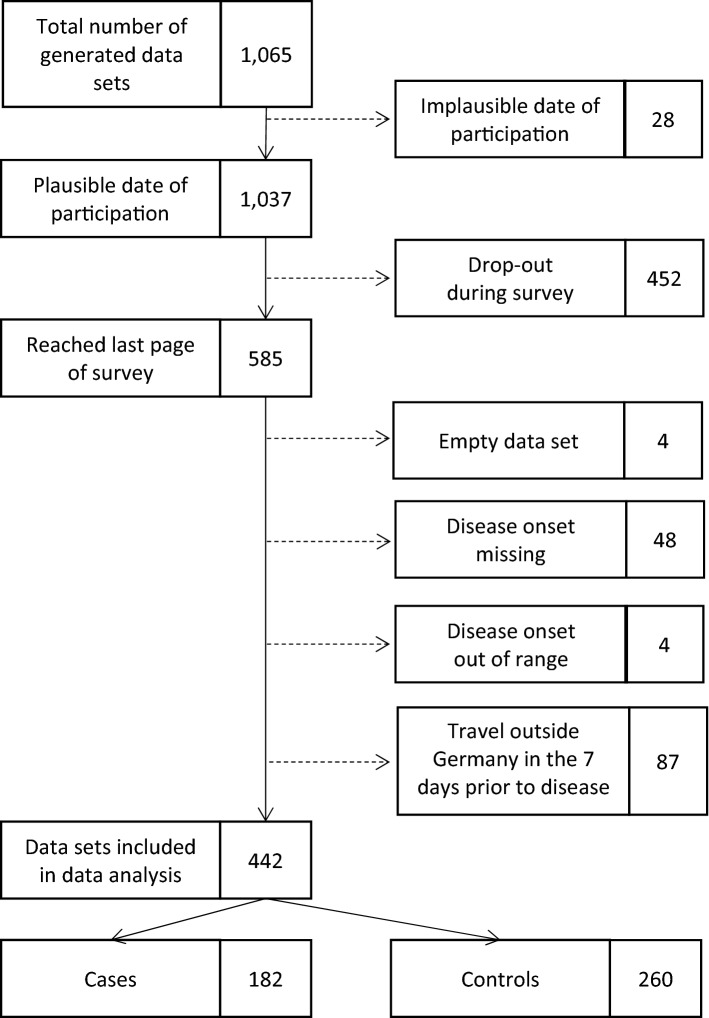
Criteria for inclusion and exclusion of questionnaire data sets in study analysis (number of data sets in boxes on the right side).

We received a total of 1065 data sets from the online survey. We excluded any data sets that were created before 23 January 2019 (n = 28), empty (n = 4), or incomplete (n = 452). Forty-eight participants did not report a day of disease onset and, therefore, could not be classified as cases or controls for the case–control study. Four participants were excluded because the reported date of disease onset was outside the study period. Eighty-seven data sets were excluded (37 cases, 50 controls) because the participants reported traveling outside of Germany in the 7 days before disease onset. Of the remaining 442 data sets, 182 were categorised as case data sets (“case-patients”) and 260 as control data sets (“control-patients”) (ratio 1:1.4) (Fig. 2). Case-patients were slightly older than control-patients (median (50th percentile) age: 48 years vs. 45 years). The mean (arithmetic mean) age of case-patients: (45.8 years; 95% CI 43.1–48.5 years) and control-patients (42.0 years; 95% CI 39.6–44.4 years) differed statistically significantly (*P*-value = 0.04). The proportion of males was higher in the case-patient group than in the control-patient group (56% vs. 49%) (Table 1). Case-patients and control-patients did not differ considerably regarding the federal state of their residency. Most case-patients (33%) and control-patients (26%) lived in North Rhine-Westphalia, the federal state in Germany with the largest population (Table 1). The subgroup of case-patients that reported disease onset within 7 days after Christmas Eve (25–31 December; “Christmas Eve cases”) consisted of 92 cases. The subgroup of case-patients that reported disease onset within 7 days after New Year’s Eve (1–7 January; “New Year’s Eve cases”) consisted of 80 cases.

**Table 1:** Characteristics of case–control study participants; *Campylobacter* enteritis winter peak, Germany 2018/2019.

Characteristics	Case-patients^a^	Control-patients^b^
Total	n = 182	n = 260
**Sex**		
Male	56.3% (n = 102)	48.4% (n = 124)
Female	43.7% (n = 79)	51.6% (n = 132)
Median age	48 years	45 years
Age range	1–86 years	0–84 years
**Age group**		
0–14 years	5.1% (n = 9)	5.7% (n = 14)
15–59 years	71.9% (n = 128)	74.7% (n = 183)
60 years or older	23.0% (n = 41)	19.6% (n = 48)
**Federal state of residency**		
Schleswig Holstein	5.6% (n = 9)	5.7% (n = 13)
Hamburg	4.3% (n = 7)	3.1% (n = 7)
Lower Saxony	9.9% (n = 16)	7.0% (n = 16)
Bremen	1.2% (n = 2)	0.4% (n = 1)
North Rhine-Westphalia	34.6% (n = 56)	26.3% (n = 60)
Hesse	2.5% (n = 4)	2.2% (n = 5)
Rhineland Palatinate	0.6% (n = 1)	0.0% (n = 0)
Baden Wuerttemberg	9.3% (n = 15)	12.3% (n = 28)
Bavaria	11.1% (n = 18)	15.4% (n = 35)
Saarland	1.9% (n = 3)	2.2% (n = 5)
Berlin	3.7% (n = 6)	3.1% (n = 7)
Brandenburg	3.7% (n = 6)	2.2% (n = 5)
Mecklenburg-Western Pommerania	1.9% (n = 3)	3.5% (n = 8)
Saxony	8.6% (n = 14)	11.4% (n = 26)
Saxony Anhalt	0.6% (n = 1)	1.8% (n = 4)
Thuringia	0.6% (n = 1)	3.5% (n = 8)

^a^Case-patients: *Campylobacter* enteritis cases with disease onset between 25 December 2018 and 8 January 2019.

^b^Control-patients: *Campylobacter* enteritis cases with disease onset between 30 November and 24 December 2018, or between 9 January and 28 February 2019.

### Response rate

The total number of *Campylobacter* cases that were notified to the local health authorities in the 15 participating federal states between 2 January and 28 February 2019 was 8278. Assuming that all potential study participants were informed about our study by their respective local health authority, 13% (1037) of the 8278 potential participants created datasets (complete or incomplete) in our online survey. The calculated overall response rate was lower (7%) because we only took the 581 data sets into account, where participants had looked at the questions up to the end of the questionnaire and at least answered some of them (“complete” data sets). We could not conduct calculations according to federal state because 51% of the 1037 study participants did not provide information regarding their postal code, which could have been used to identify the federal state they lived in.

The 390 case- and control-patients included in our study, where the federal state of residency could be deduced from the given postal code (88% of the 442 cases and controls included in the case–control study), represented 10% of all *Campylobacter* cases that were notified to the 15 participating federal state health authorities with information on the date of disease onset, and that would have fulfilled the inclusion criteria for our case–control study (n = 3903). This proportion varied by federal state from 4 to 41%.

### Clinical aspects

Case-patients and control-patients did not differ significantly with respect to self-reported symptoms. Therefore, we analysed both groups together. The frequencies of reported gastrointestinal symptoms were as follows: 97% diarrhoea, 86% abdominal pain, 78% liquid stool, 28% visible blood in stool, 20% vomiting. Diarrhoea lasted for a median of 7 days (interquartile range (IQR 25th–75th percentile) 5–12 days). About half of the patients reported fever (53%). Weakness/fatigue was reported by 93%, pain in the joints by 62%, and headache by 55%. When asked for the reasons why they had sought medical attention, 78% of patients answered that the symptoms were severe, and/or did not improve (75%). About half of the patients (47%) visited a doctor’s office because they needed an incapacity certificate for work. About 17% of patients were treated in a hospital because of their *Campylobacter* infection, for a median of 4 days (IQR 3–5 days). About a quarter of patients (26%) reported that they had been prescribed one or more antibiotics for treatment of the *Campylobacter* infection. The most frequently named antibiotics were azithromycin (30%) and ciprofloxacin (28%). About one quarter (28%) of patients who were treated with an antibiotic could not name it.

### Consumption of meat fondue meals on the Christmas and New Year’s holidays

Of all 442 study participants that were included in data analyses, 16% (n = 71) reported that they had had a fondue meal on the Christmas or New Year’s holidays (23% of case-patients, 13% of control-patients). Of those, the vast majority (n = 64; 90%) reported that meat had been offered at the fondue meal. A meat fondue meal was more popular on the Christmas than on the New Year’s holidays (10% vs. 6% of all participants). The most popular date for having a fondue meal was 24 December (47% of all participants who reported meat fondue consumption on the holidays), followed by New Year’s Eve (31 December; 36%). Of the 4 types of meat that we queried about, beef (92%) was most frequently offered, followed by pork (72%), chicken (63%), and turkey (44%). At 97% of the 64 meat fondue events, more than one type of meat was offered, most frequently 3 types of meat (56%), followed by 2 types of meat (33%). When 3 types of meat were offered the most popular combination was chicken, pork, and beef (58%); when 2 types were offered chicken and beef was the most popular combination (43%). The meat was mainly heated in oil (68%), rather than in a broth (30%). A large proportion of participants (80%) of meat fondue meals reported that they had touched the raw meat with their bare fingers, 30% reported that raw and cooked meat had been placed on the same plate, and 94% reported that food items that remained uncooked, e.g., salads, dips, were offered at the meat fondue meal. The vast majority (94%) reported that they had had the meat fondue meal in a private household, only 2% had had the meat fondue meal in a restaurant.

The median number of people participating at the meat fondue meals was 5 (range 2–18; IQR 2–12). Of the 38 case-patients who reported that they had had a meat fondue meal at Christmas (n = 22), the New Year’s holidays (n = 11) or both (n = 5), 30 (79%) had a disease onset date in the following 7 days. The other 8 case-patients became ill more than 7 days after the meat fondue meals (n = 5), on the same day they had the meat fondue meal (n = 1), before the meat fondue meal (n = 1), or the exact date of the meat fondue meal remained unclear (n = 1).The 30 case-patients with disease onset within the 7 days after the meat fondue meals reported a total of at least 150 attendees (data missing from one case-patient), of which 35 (23%) had gastrointestinal symptoms afterwards, including the 30 case-patients. It remains unknown if the additional 5 persons who fell ill afterwards also had a *Campylobacter* infection, as they were not notified as such.

A total of 26 control-patients reported that they had had meat fondue meals on the holidays with a total of 163 attendees of whom one person (0.6%), not a control-patient but another person, became ill with gastrointestinal symptoms within the 7 days afterwards. It is unknown if the fondue participant’s gastrointestinal symptoms resulted from a *Campylobacter* infection.

### Consumption of raclette grill meals with meat on the Christmas and New Year’s holidays

Of all 442 study participants that were included in the data analyses, 32% (n = 142) reported that they had had a raclette grill meal on the Christmas or New Year’s holidays (36% of case-patients, 29% of control-patients). Meat had been offered at most of the raclette grill meals (n = 116; 82%). The most popular date for raclette grill meals was New Year’s Eve (53% of all participants who reported a raclette grill meal on the holidays), followed by Christmas Eve (24%). The most frequently offered type of meat at raclette grill meals was beef (72%), followed by pork (63%), chicken (55%), and turkey (41%). At 72% of raclette grill meals (n = 83), more than one type of meat was offered, most frequently 3 types (37%) or 2 types (25%). When 3 types of meat were offered, chicken, pork and beef was the most frequent combination (49%), followed by turkey, pork, and beef (37%). When 2 types of meat were offered, turkey and beef (28%), and chicken and beef (24%) were most popular.

Less than half of all study participants (40%) reported that they had touched the raw meat with their bare fingers, and 15% reported that raw and cooked meat had been placed on the same plate. Eighty-five per cent of participants reported that food items that remained uncooked, such as salads or dips, had been offered along with the meat. All raclette grill meals on the holidays had taken place in private households.

A median of 6 persons attended the raclette grill meals (range 2–18 persons; IQR 3–12 persons). Of the 55 case-patients who reported that they had had a raclette grill meal with meat at Christmas (n = 12), the New Year’s holidays (n = 37) or both (n = 6), 45 (82%) had a disease onset date in the following 7 days. The other 10 case-patients became ill more than 7 days after the raclette grill meal (n = 4), on the same day (n = 2) or before (n = 3) they had the raclette grill meal, or the exact date of the meat fondue meal remained unclear (n = 1). The 45 case-patients with disease onset in the 7 days after the raclette grill meal reported a total of at least 256 attendees (data missing for 2 case-patients). In addition to the 45 case-patients 10 other raclette grill meal attendees became ill with gastrointestinal symptoms afterwards (total: n = 55; 21%). Sixty-one control-patients had a raclette grill meal with meat on the holidays with a total of at least 371 attendees. Of those, 10 persons (3%), not control-patients but other attendees, had gastrointestinal symptoms within the 7 days afterwards. It remains unknown if the gastrointestinal symptoms of the other raclette grill meal attendees were caused by *Campylobacter* infections.

### Consumption of other tabletop grill meals on the Christmas and New Year’s holidays

A much smaller number of study participants (4%; n = 16) reported that they had attended meals, where meat was heated directly at the table, other than fondue or raclette grill meals (e.g., with a tabletop grill, hot stone or “Korean barbecue”) (4% of case-patients and 4% of control-patients). The most popular dates for these types of meals were 31 December (60%) and 24 December (38%). The most frequently offered type of meat was beef (80%), followed by pork (67%), chicken (64%), and turkey (56%). Fifty per cent of all study participants who had attended this type of meal had touched the raw meat with their bare fingers, 31% reported that raw and cooked meat had been placed on the same plate, and 75% reported that food items that remained uncooked had been offered at that meal. The majority of participants had attended these meals in private households (88%; n = 14), 2 persons (12%) reported consumption in a restaurant. The median number of attendees was 4 (range 2–15; IQR 4–7). Because the number of case- and control patients who reported consumption of tabletop grill meals was small, we did not analyse this type of exposure in detail and focussed on fondue and raclette grill meals as relevant exposures on the holidays.

### Single variable analyses

Single variable analyses (adjusted for age group and sex) showed a statistically significant association of consumption of a meat fondue or a raclette grill or another tabletop grill meal on the holidays and a *Campylobacter* infection after the holidays (aOR 2.3; 95% CI 1.5–3.5). When the variables were analysed individually, the association was strongest for consumption of meat fondue (aOR 2.2; 95% CI 1.2–3.8), compared with consumption of a raclette grill meal with meat (aOR 1.5; 95% CI 1.0–2.4), and consumption of another type of tabletop grill meal (aOR 1.1; 95% CI 0.4–2.9) on the holidays. Poultry meals, other than meat fondue/raclette grill/other tabletop grill meals, consumed on the Christmas or New Year’s holidays were not associated with *Campylobacter* enteritis with disease onset after the holidays (Table 2).

**Table 2:** Single variable logistic regression analysis (adjusted for sex and age group) of case-patients (n = 182) and control-patients (n = 260) regarding exposure to certain meals on the Christmas and New Year’s holidays; case-control study, Germany, 2018/2019.

Exposure on holidays^a^		Case-patients^b^ % (n)	Control-patients^c^ % (n)	Adjusted OR^d^	95% CI^e^
Meat fondue	Any meat	21.1 (38)	10.1 (26)	**2.2**	1.2–3.8
	Chicken	14.4 (26)	5.4 (14)	**2.7**	1.4–5.5
	Turkey	8.4 (15)	4.7 (12)	1.7	0.8–3.8
	Pork	15.0 (27)	7.4 (19)	**2.1**	1.1–4.0
	Beef	19.4 (35)	9.3 (24)	**2.1**	1.2–3.8
Raclette grill meal	Any meat	30.6 (55)	23.8 (61)	1.5	1.0–2.4
	Chicken	19.9 (35)	10.2 (26)	**2.3**	1.3–4.1
	Turkey	12.9 (23)	9.1 (23)	1.5	0.8–2.8
	Pork	15.6 (28)	17.3 (44)	1.0	0.6–1.6
	Beef	21.2 (38)	16.7 (42)	1.5	0.9–2.5
Other tabletop grill meal	Any meat	1.1 (2)	1.9 (5)	0.5	0.1–2.7
	Chicken	2.8 (5)	1.5 (4)	1.7	0.5–6.6
	Turkey	2.3 (4)	1.9 (5)	1.1	0.3–4.1
	Pork	1.7 (3)	2.7 (7)	0.6	0.2–2.3
	Beef	2.8 (5)	2.7 (7)	0.9	0.3–3.0
Poultry meal	Other than fondue/raclette grill/table grill	45.2 (33)	44.8 (64)	1.0	0.6–1.8

^a^Christmas holidays (24–26 December) or New Year holidays (31 December–1 January).

^b^Case-patients: Disease onset 25 December 2018–8 January 2019.

^c^Control-patients: Disease onset 30 November–24 December 2018, or 9 January–28 February 2019.

^d^Odds ratio (adjusted for age group and sex). Statistically significant odds ratios (*P*-values ≤ 0.05) are depicted in bold.

^e^Confidence interval.

When we looked at the types of meat that were offered at the meat fondues on the holidays, a positive association with *Campylobacter* enteritis after the holidays was found for chicken meat (aOR 2.7; 95% CI 1.4–5.5), but also for pork (aOR 2.1; 95% CI 1.1–4.0) and beef (aOR 2.1; 95% CI 1.2–3.8) (Table 2). We found no statistically significant positive association of *Campylobacter* enteritis and the queried behaviours during meat fondue meals (touching raw meat with bare fingers, placing raw and cooked meat on the same plate, or serving raw meat and food items that remained uncooked at the same meal) (Table 3). Regarding raclette grill meals consumed on the holidays, we found a positive association of *Campylobacter* enteritis after the holidays with chicken meat only (aOR 2.3; 95% CI 1.3–4.1) (Table 2). None of the queried behaviours during the raclette grill meals were statistically significantly associated with being a case (Table 3).

**Table 3:** Single variable logistic regression analysis (adjusted for sex and age group) of case-patients (n = 182) and control-patients (n = 260) regarding risk behaviour during holiday meals; case-control study, Germany, 2018/2019.

Risk behaviour during holiday^a^ meal		Case-patients^b^ % (n)	Control-patients^c^ % (n)	Adjusted OR^d^	95% CI^e^
Meat fondue (N = 64)	Raw meat touched with bare fingers	81.6 (31)	76.9 (20)	1.2	0.3–4.2
	Raw and cooked meat placed on the same plate	26.3 (10)	36.0 (9)	0.6	0.2–2.0
	Uncooked food items offered^f^	94.7 (36)	92.3 (24)	1.4	0.2–11.0
Raclette grill meal (N = 116)	Raw meat touched with bare fingers	29.8 (14)	48.3 (28)	0.5	0.2–1.1
	Raw and cooked meat placed on the same plate	18.4 (9)	12.3 (7)	1.6	0.5–5.1
	Uncooked food items offered^f^	88.5 (46)	81.4 (48)	1.9	0.6–5.9
Other tabletop grill meal (N = 16)	Raw meat touched with bare fingers	42.9 (3)	55.6 (5)	0.8	0.1–6.4
	Raw and cooked meat placed on the same plate	14.3 (1)	44.4 (4)	0.1	0.0–1.6
	Uncooked food items offered^f^	57.1 (4)	88.9 (8)	0.2	0.0–2.4

^a^Christmas holidays (24–26 December) or New Year holidays (31 December–1 January).

^b^Case-patients: Disease onset 25 December 2018–8 January 2019.

^c^Control-patients: Disease onset 30 November–24 December 2018, or 9 January–28 February 2019.

^d^Odds ratio (adjusted for age group and sex).

^e^Confidence interval.

^f^Salad, dips, etc.

In further analyses, we looked at subgroups of cases depending on their date of disease onset after the Christmas or New Year’s holidays (“Christmas Eve cases” and “New Year’s Eve cases”). We found a positive association between *Campylobacter* enteritis with disease onset after Christmas and meat fondue consumed at Christmas (aOR 3.5; 95% CI 1.7–7.2), in particular with chicken meat fondue (aOR: 6.1; 95% CI 1.0–36.6). Raclette grill meals with meat consumed at Christmas were not associated with *Campylobacter* enteritis with disease onset after Christmas (aOR 1.1; 95% CI 0.5–2.3). *Campylobacter* enteritis with disease onset after the New Year’s holiday was positively associated with meat fondue meals (aOR 3.1; 95% CI 1.3–7.5) as well as raclette grill meals with meat (aOR 3.9; 95% CI 2.1–7.0). Due to the small number of cases in the subgroups, further analyses were not conducted.

In an analysis of food items consumed in the 7 days prior to disease onset, we detected positive associations between the types of meat that are typically consumed on the holidays (goose, duck, turkey, but also beef and pork) and *Campylobacter* enteritis after the holidays, because the frequency of consumption of these types of meats was higher in the case-patient group (the 7-day time period before disease onset included the holidays) than in the control-patient group (the 7-day time period before disease onset did not include the holidays) (Table 4).

**Table 4:** Single variable logistic regression analysis (adjusted for sex and age group) of case-patients (n = 182) and control-patients (n = 260) regarding exposure to certain food items in the 7 days before disease onset; case-control study, Germany, 2018/2019.

Consumed in the 7 days before disease onset	Case-patients^a^ % (n)	Control-patients^b^ % (n)	Adjusted OR^c^	95% CI^d^
Chicken	65.7 (111)	69.6 (160)	0.8	0.5–1.3
Turkey	28.7 (50)	17.3 (40)	**1.9**	1.2–3.1
Duck	18.2 (32)	8.1 (20)	**2.4**	1.3–4.4
Goose	16.4 (29)	2.5 (6)	**7.2**	2.9–17.9
Pork	55.2 (90)	44.1 (97)	**1.6**	1.0–2.4
Beef	48.3 (84)	27.4 (60)	**2.4**	1.6–3.7
Lamb	8.6 (15)	5.7 (14)	1.5	0.7–3.1
Ground pork and beef (mixed)	26.7 (43)	27.6 (61)	1.0	0.6–1.6
Ground pork	15.3 (24)	25.6 (57)	**0.5**	0.3–0.9
Ground beef	19.3 (31)	21.7 (47)	0.9	0.5–1.5
Ground poultry	11.0 (18)	10.5 (24)	1.1	0.5–2.1
Unpasteurised milk	7.2 (13)	5.1 (13)	1.4	0.6–3.1
Meal outside own household	61.1 (110)	69.2 (171)	**0.7**	0.4–1.0

^a^Case-patients: Disease onset 25 December 2018–8 January 2019.

^b^Control-patients: Disease onset 30 November–24 December 2018, or 9 January–28 February 2019.

^c^Odds ratio (adjusted for age group and sex). Statistically significant odds ratios (*P*-values ≤ 0.05) are depicted in bold.

^d^Confidence interval.

### Dates of disease onset among control-patients and sensitivity analysis

By definition, none of the 260 control-patients had a disease onset date within 7 days after the holidays. We analysed their dates of disease onset in more detail. For a large majority of control-patients (85%) disease onset was on or after 9 January 2019. Of the 39 control-patients (15%) with a date of disease onset in December 2018, 11 became ill in the time period 1–18 December, and 28 became ill in the time period 19–24 December 2018, that is, within the 7 days immediately before Christmas. Of the 207 control-patients who did not have a meat fondue or a raclette grill meal on the holidays, 22 (11%) had a disease onset date between 19 and 24 December 2018, which may have been a reason for not having a fondue or raclette meal on the holidays. Of those, 10 did not report any other food consumption. Assuming that 2 of them would have had a meat fondue meal and 3 of them would have had a raclette grill meal on the holidays if they had not been ill immediately before the holidays, we re-calculated the adjusted odds ratios (fondue with any meat: aOR 2.0; 95% CI 1.2–3.4; raclette grill meal with meat: aOR 1.4, 95% CI 0.9–2.2) (Table 2).

## Discussion

In the present study, we analysed the annually recurring increase of notified *Campylobacter* enteritis cases after the holidays. The results of our case–control study confirmed our hypothesis that meat fondue or raclette grill meals consumed on the Christmas or New Year’s holidays are risk factors for *Campylobacter* enteritis with disease onset in late December or early January. Furthermore, chicken meat consumed at the fondue or raclette meals was positively associated with disease onset after the holidays. This is in line with results from a case–control study conducted in Switzerland that found a positive association of consumption of meat fondue in the festive season and winter peak *Campylobacter* enteritis, especially when chicken meat was served at the fondue^8^.

It has been demonstrated in numerous case–control and source attribution studies to date that consumption of chicken meat is the most important risk factor and chicken the most important source for sporadic *Campylobacter* infections in many European and non-European countries^11–21^.

In our study, the association of winter peak *Campylobacter* enteritis with meat fondue involving chicken meat was slightly stronger than with raclette grill meals where chicken was served. One explanation may be that during fondue meals chicken meat is handled differently than during raclette grill meals. About 80% of study participants (case- or control-patients) who had attended a meat fondue event reported that they had touched the raw meat with their bare fingers. Fresh chicken meat at retail is often contaminated with *Campylobacter* in Germany. The prevalence of *Campylobacter* was about 50% in fresh broiler meat samples for zoonosis monitoring in Germany^22–24^. Hence, it is likely that by touching the raw meat, contamination of fingers and subsequent cross-contamination of other food items may have occurred. Study participants who had consumed a raclette grill meal reported touching the raw meat with their bare fingers at a much lower frequency. In addition, raw and cooked meat items were placed on the same plate at higher frequency during meat fondues than during raclette grill meals. When we analysed the risk behaviour at fondue or raclette grill meals, such as touching the meat with bare fingers or placing raw and cooked meat on the same plate, using logistic regression, we did not find statistically significant associations with *Campylobacter* enteritis with disease onset after the holidays. In contrast, in the case control-study conducted in Switzerland, the risk of *Campylobacter* infection was significantly lowered if raw and cooked meat were placed on separate plates during a meat fondue meal (multivariable odds ratio (mOR): 0.2), or if a plate with compartments was used (mOR: 0.4)^8^. We may not have been able to detect this effect in our study, because the total number of case-patients and control-patients who had attended a meat fondue meal was smaller (74 cases and 75 controls in the Swiss study vs. 38 cases and 26 controls in this study), and, in addition, data regarding behaviour during meat fondue meals was not provided by all meat fondue participants in our study (Table 3).

Meat fondues or raclette grill meals where turkey meat was offered were not positively associated with *Campylobacter* enteritis with disease onset after the holidays. One reason may be that turkey meat was the least popular meat at fondue and raclette grill meals. Also, in comparison to chicken meat, turkey meat is not as highly contaminated with *Campylobacter*. Data from the zoonosis monitoring in Germany showed that 19% of samples of fresh turkey meat from conventional farming tested positive^23^. Consumption of turkey meat was not a statistically significant risk factor for *Campylobacter* infections in our previous case–control study^5^.

In addition to the association with fondue meals with chicken meat, we also detected positive associations with fondue meals with beef or pork and *Campylobacter* enteritis with disease onset after the holidays. One explanation may be that at the meat fondue meals more than one type of meat was offered, with pork, beef and chicken as a popular combination. This resulted in a correlation of the chicken meat variable with the variables for beef and pork. While it cannot be excluded that consumption of contaminated beef and pork may also lead to *Campylobacter* infections, our results, in combination with results from previous studies^11–21^, point toward chicken meat consumed at the fondue meals as the most likely food vehicle for *Campylobacter* infections with disease onset after the holidays.

Interestingly, we saw a continuous decrease of winter season *Campylobacter* enteritis cases in the past years up to the year when we conducted our study. This was in line with the general decrease in annually reported *Campylobacter* cases since 2016, but may also have been caused by a decreasing popularity of fondue and raclette grill meals on the holidays, or with an increase of hygiene measures taken during these meals over the years. In our study, Christmas was the most popular time for consumption of meat fondue meals, whereas New Year’s Eve was the most popular date for consumption of raclette grill meals. Overall, about 16% of our study participants reported fondue meal consumption, and 32% reported raclette grill meal consumption on the holidays. The frequency of fondue consumption in our study was similar to surveys regarding popular meals on Christmas or New Year’s Eve that were found on the internet, whereas the frequency of raclette grill meal consumption was higher in our study. About 17% of participants of a survey conducted in 2020 reported consumption of fondue or raclette on Christmas Eve^25^. In a survey from 2014, 16% reported raclette grill meal consumption and 16% reported fondue meal consumption on New Year’s Eve^26^. However, it should be kept in mind that the quality of the surveys found on the internet could not be assessed in any detail, and that they can only serve as a rough benchmark at best. We could not find any data on consumption of fondue or raclette grill meals on the holidays in Germany that were published in peer-reviewed journals.

In our study, we used a method where reported *Campylobacter* enteritis cases served as case-patients as well as control-patients depending on their dates of disease onset. This approach, which has advantages but also limitations, was chosen for several reasons. We had already identified the most important risk factors for sporadic *Campylobacter* enteritis in Germany in general, not only with disease onset after the holidays, in a previous case–control study with controls from the general population^5^, and wanted to test our hypotheses regarding fondue and raclette grill meals consumed on the holidays in the present study. As notified *Campylobacter* enteritis cases, both case- and control-patients could be recruited through local health authorities. The recruitment of controls through the local health authorities was viewed as a more convenient and faster approach than finding suitable control persons in the general population. Furthermore, some potential study biases (e.g., selection bias, recall bias) can be mitigated with an approach that compares cases to other cases^27,28^. For some aspects of our study, both groups could be analysed together, e.g., with respect to symptoms and severity of disease. We assumed that both case-patients and control-patients, regardless of their dates of disease onset, would be able to remember equally well what they had eaten on the Christmas and New Year’s holidays because these days are considered as special by many people in Germany.

We may have introduced a bias and over-estimated the association with meat fondue or raclette grill meal consumption due to our study design because control-patients with disease onset on the days immediately before Christmas may not have had a meat fondue or raclette meal on the holidays because of *Campylobacter* enteritis symptoms they may have had on these days. Therefore, we analysed their dates of disease onset in more detail. Only about 10% of control-patients who did not have a fondue or raclette meal on the holidays had a disease onset on the days before Christmas. If their disease onset had been before the New Year’s holidays they would have been classified as case-patients. More than half of those (12/22) reported other meals they had eaten on the holidays, so obviously they were well enough to eat and theoretically could have consumed food at a meat fondue or raclette grill meal. For the other 10 control-patients information on the food they had consumed on the holidays was not available. We assumed that they were too ill to eat holiday meals and that a certain proportion of them may have had a fondue or raclette grill meal if they had they not been ill. The results did not change substantially; the calculated adjusted odds ratios were only slightly smaller (meat fondue (any meat): OR 2.0 instead of 2.2; raclette grill meal (any meat): OR 1.4 instead of 1.5).

As expected, due to our study design, analysing exposure to various food items in the 7 days before disease onset did not give meaningful results with regard to identification of risk factors for *Campylobacter* infections after the holidays. By definition, the Christmas and New Year holidays fell within the 7-day time period before disease onset of case-patients. Food items that are frequently consumed on the holidays in general, e.g., goose (“Christmas goose”) and duck, were reported more frequently by case-patients than by control-patients, thus resulting in a positive association with being a case. Therefore, we did not consider food items consumed in the 7 days prior to disease onset as “real” risk factors for *Campylobacter* infections after the holidays even if they were positively associated. Nevertheless, we chose to ask about several food items that are known or suspected risk factors for *Campylobacter* enteritis in general, such as chicken meat, ground pork, or unpasteurized milk, because we feared that study participants, especially those with dates of disease onset other than late December or early January, would be puzzled if we only asked them about food consumption on the holidays and, consequently, not complete the questionnaire.

Interestingly, chicken meat was consumed at about the same frequency by case- and control-patients in the 7 days before disease onset. This indicated that chicken was not a type of meat preferably consumed on the Christmas or New Year’s holidays.

Our study design had some limitations but was appropriate for the hypotheses we wanted to analyse. Repeating the study using a different study design (e.g., comparing *Campylobacter* enteritis cases with controls from the general population) would allow us to examine additional aspects regarding risk factors for *Campylobacter* enteritis with disease onset after the holidays.

## Conclusions

The annual peak of *Campylobacter* enteritis cases after the Christmas and New Year holidays may be caused, at least partially, by consumption of meat fondues and raclette grill meals on the holidays. At these types of meals raw meat is typically handled directly at the table and touched with bare fingers, in particular at fondue meals. The odds of infection are higher when chicken meat is offered at these meals, because chicken meat is often contaminated with *Campylobacter*.

In Germany, consumer recommendations regarding food safety are given by the Federal Institute for Risk Assessment (Bundesinstitut für Risikobewertung, BfR), an institute within the portfolio of the Federal Ministry of Food and Agriculture. In 2015, recommendations regarding the safety of meat fondues were published by the BfR^29^. Recommendations include good kitchen hygiene, such as the strict separation of raw meat and other food items that are consumed uncooked; and thoroughly washing hands, kitchen utensils and surfaces after they have come into contact with food items of animal origin and before preparation of other food items. To prevent *Campylobacter* infections after the holidays in the future, consumers should be made aware each year before the Christmas and New Year holidays about these recommendations and the infection risks they may encounter when participating in meat fondue or raclette grill meals, especially if chicken meat is offered at these meals.

## Supplementary Information

Supplementary Information.

## Data Availability

Datasets generated and analysed during the current study will be made available upon reasonable request to first or last author.
