# Supplementary material for: Investigating the Campylobacter enteritis winter peak in Germany, 2018/2019
Source: Sci Rep. 2021 Nov 25;11:22902. doi: 10.1038/s41598-021-02423-8 (PMC8617151; doi:10.1038/s41598-021-02423-8)
Supplement: Supplementary file 1 — Supplementary Information. [file 41598_2021_2423_MOESM1_ESM.pdf]

ROBERT KOCH INSTITUT

Study to determine risk factors for *Campylobacter* infections between December and February

Online Questionnaire
for case-control study conducted
between 21 January and 18 March 2019; Germany
(Robert Koch-Institute, Berlin, Germany)

[Translated from German]

Introduction

The Robert Koch Institute in Berlin is currently conducting a scientific study regarding infections with the bacterium *Campylobacter*. With our study we would like to find out why an increase of *Campylobacter* infections can be observed in the winter.

We would like to kindly ask persons who have contracted a *Campylobacter* infection in the past weeks, or whose children contracted a *Campylobacter* infection, to provide some information about their consumption of food items in the days before disease onset and on the holidays (Christmas, New Year's Eve; New Year).

We would be pleased if you agreed to participate in our survey. The survey takes about 10 to 20 minutes. In order for the study to deliver meaningful results, it is very important to fill out the questionnaire carefully and as completely as possible.

Your participation in the survey is of course voluntary. Your data will be treated confidentially and will not be passed on to third parties. Your data is collected anonymously and is only evaluated and published by the Robert Koch Institute in a summarized form, i.e., without any reference to you. If you do not want to participate, there will be no disadvantages for you. You can also cancel the survey at any time or not answer individual questions.

If it was your child and not you who was ill, we would ask you to answer the questions for your child or together with your child. If more than one child in your household was infected with *Campylobacter* in the past few weeks, please only answer the questions for the child who contracted the infection first.

Participation in the study by the Robert Koch Institute is independent of the investigations by your local health authority. Please also answer the questions your local health authority may have about your illness.

The data protection officer of the Robert Koch Institute (contact: 030-18754-xxxx [*DELETED*]; Datenschutz@rki.de) has evaluated our study plan. By participating, you consent that your data or your child's data can be used for the purposes described.

○ Yes, I agree and will participate.

○ No, I do not want to participate.

1	**Have you had a *Campylobacter* infection yourself or do you answer the questions for your sick child?**<br><br>☐ I was/am sick myself<br><br>☐ I am answering the questions for a sick child<br><br>*[If answering for a sick child, questions were rephrased accordingly in the online questionnaire.]*
2	**Illness**<br>First of all, we would like to ask you about the symptoms of your *Campylobacter* infection.
2.1	**Which of the following symptoms did you experience?**

	Yes	No	Don't know	Don't want to answer
Diarrhoea (i.e.,3 or more unformed stools in 24 hours)	☐	☐	☐	☐
Liquid and / or mushy stool (fewer than 3 unformed stools in 24 hours)	☐	☐	☐	☐
Visible blood in the stool	☐	☐	☐	☐
Abdominal pain	☐	☐	☐	☐
Fever (i.e., body temperature higher than 38.5°C)	☐	☐	☐	☐
Vomiting	☐	☐	☐	☐
Headache	☐	☐	☐	☐
Pain in the joints/limbs	☐	☐	☐	☐
Fatigue, weakness, exhaustion	☐	☐	☐	☐

2.2	**When did the diarrhea start? If you have not had diarrhea, when did the first symptoms start?**<br>Please give the date as precisely as possible.<br><br>___ ___ _____ ☐ I cannot remember the exact date.<br>Day Month Year ☐ I do not want to answer this question.
2.2.1	*If „I cannot remember the exact date" was chosen:*<br><br>**Perhaps you can narrow down the time period of the onset of your illness.**<br>**Was the onset of the illness likely...:**<br><br>☐ before or on 24 December 2018 (Christmas Eve)?<br><br>☐ after 24 December 2018 (Christmas Eve), but before Wednesday, 9 January 2019?<br><br>☐ on or after Wednesday, 9 January 2019?<br><br>☐ I do not remember.<br><br>☐ I do not want to answer this question.
2.3	**On how many days in total did you have diarrhoea? If you did not have diarrhoea, how long did the symptoms last?**<br><br>___ Days ☐ Don't know

**2.4**	**Why did you seek medical treatment?** Multiple answers are possible.

I had severe symptoms, e.g. severe abdominal pain, severe diarrhoea, bloody diarrhoea.	☐
The symptoms did not improve.	☐
I needed an incapacity certificate for work.	☐
I had another reason.	☐
I do not remember.	☐
I do not want to answer this question.	☐

2.4.1 *If „incapacity certificate for work":*

For how many working days did you have to take sick leave?

└─┴─┘ Working days ☐ I do not remember.

☐ I do not want to answer this question.

2.4.1.1 *If any answer of Q2.4.1:*
Are you still unable to work today because of your *Campylobacter* infection?

☐ Yes ☐ No ☐ Don't know ☐ I do not want to answer this question.

2.5 **Did you take an antibiotic because of your *Campylobacter* infection?**

☐ Yes ☐ No ☐ Don't know ☐ I do not want to answer this question.

2.5.1 *If Yes at Q2.5:*

Have you taken any of the following antibiotics because of your *Campylobacter* infection?

Azithromycin (e.g., Azithrobeta®, Zithromax®)	☐
Ciprofloxacin (e.g., Ciprobay®, Ciprobeta®, Cipro-1A Pharma®)	☐
Erythromycin (e.g., Eryhexal®, Infectomycin®)	☐
Clarithromycin	☐
Metronidazol	☐
A different antibiotic	☐
…namely: ____________________	
I do not know which antibiotic I was taking.	☐
I do not want to answer this question.	☐

**2.6**	**Have you been hospitalised for your *Campylobacter* infection?**<br><br>☐ Yes ☐ No ☐ I do not want to answer this question.
**2.6.1**	*If Yes at Q2.6:*<br>**How long were you in the hospital for your *Campylobacter* infection?**<br>Please indicate the number of nights you stayed in the hospital.<br><br>└─┴─┘ Nights ☐ Don't know<br><br>☐ I have not spent a night in a hospital because of my *Campylobacter* infection.<br><br>☐ I do not remember.<br><br>☐ I do not want to answer this question.

**3**	**Travel**
**3.1**	**Did you travel abroad in the 7 days before the start of your illness, i.e. did you spend at least one night abroad?**<br><br>☐ Yes<br><br>☐ No<br><br>☐ I do not remember.<br><br>☐ I do not want to answer this question.

4	***Consumption of meat*** <br>**Did you prepare or eat minced meat in the 7 days before the onset of your illness?** Please state in the respective lines whether you prepared minced meat and whether you ate minced meat.
**4.1.**	**Minced meat**

4.1.1

**Mixed minced pork and beef**	Prepared<br>☐ Yes<br>☐ No<br>☐ Don't know		Ate<br>☐ Yes<br>☐ No<br>☐ Don't know	
If „Prepared":<br>**Did you touch the raw mixed minced meat with your fingers?**	☐ Yes	☐ No	☐ Don't know	
If „Ate":<br>**Was the mixed minced meat that you ate…**<br>*(WITH PICTURES)*	☐ raw	☐ partially raw (not completely cooked)	☐ completely cooked	☐ Don't know
If „Prepared" or „Ate":<br>**Before preparation, was the mixed minced meat …**<br>*(WITH PICTURES)*	☐ fresh, e.g. from the butcher or from the meat counter	☐ packaged, e.g. from the refrigerated shelf	☐ frozen (frozen or self-frozen mixed minced meat)	☐ Don't know

4.1.2

**Minced pork**	Prepared<br>☐ Yes<br>☐ No<br>☐ Don't know		Ate<br>☐ Yes<br>☐ No<br>☐ Don't know	
If „Prepared":<br>**Did you touch the raw minced pork with your fingers?**	☐ Yes	☐ No	☐ Don't know	
If „Ate":<br>**Was the minced pork that you ate…**<br>*(WITH PICTURES)*	☐ raw	☐ partially raw (not completely cooked)	☐ completely cooked	☐ Don't know
If „Prepared" or „Ate":<br>**Before preparation, was the minced pork…**<br>*(WITH PICTURES)*	☐ fresh, e.g. from the butcher or from the meat counter	☐ packaged, e.g. from the refrigerated shelf	☐ frozen (frozen or self-frozen minced pork)	☐ Don't know

4.1.3	**Minced beef**	Prepared<br>☐ Yes<br>☐ No<br>☐ Don't know		Ate<br>☐ Yes<br>☐ No<br>☐ Don't know	
	If „Prepared":<br>**Did you touch the raw minced beef with your fingers?**	☐ Yes	☐ No	☐ Don't know	
	If „Ate":<br>**Was the minced beef that you ate…**<br>*(WITH PICTURES)*	☐ raw	☐ partially raw (not completely cooked)	☐ completely cooked	☐ Don't know
	If „Prepared" or „Ate":<br>**Before preparation, was the minced beef…**<br>*(WITH PICTURES)*	☐ fresh, e.g. from the butcher or from the meat counter	☐ packaged, e.g. from the refrigerated shelf	☐ frozen (frozen or self-frozen minced beef)	☐ Don't know

4.1.4	**Minced poultry meat**	Prepared<br>☐ Yes<br>☐ No<br>☐ Don't know		Ate<br>☐ Yes<br>☐ No<br>☐ Don't know	
	If „Prepared":<br>**Did you touch the raw minced poultry meat with your fingers?**	☐ Yes	☐ No	☐ Don't know	
	If „Ate":<br>**Was the minced poultry meat that you ate…**<br>*(WITH PICTURES)*	☐ raw	☐ partially raw (not completely cooked)	☐ completely cooked	☐ Don't know
	If „Prepared" or „Ate":<br>**Before preparation, was the minced poultry meat…**<br>*(WITH PICTURES)*	☐ fresh, e.g. from the butcher or from the meat counter	☐ packaged, e.g. from the refrigerated shelf	☐ frozen (frozen or self-frozen minced poultry meat)	☐ Don't know

4.2	**Did you prepare or eat the following types of meat as roast, sliced meat, schnitzel, steak or similar in the 7 days before the onset of your illness?**

4.2.1

**Chicken,** e.g. chicken breast, leg, wings, whole chicken or chicken for soup	Prepared<br>☐ Yes<br>☐ No<br>☐ Don't know		Ate<br>☐ Yes<br>☐ No<br>☐ Don't know	
If „Prepared":<br>**Did you touch the raw chicken meat with your fingers?**<br>(WITH PICTURE)	☐ Yes	☐ No	☐ Don't know	
If „Ate":<br>**Was the chicken meat that you ate…**	☐ raw	☐ partially raw (not completely cooked)	☐ completely cooked	☐ Don't know
If „Prepared" or „Ate":<br>**Before preparation, was the chicken meat…**<br>*(WITH PICTURES)*	☐ fresh, e.g. from the butcher or from the meat counter	☐ packaged, e.g. from the refrigerated shelf	☐ frozen (frozen or self-frozen chicken meat)	☐ Don't know

4.2.2

**Turkey** e.g. turkey breast, leg, wings, whole turkey	Prepared<br>☐ Yes<br>☐ No<br>☐ Don't know		Ate<br>☐ Yes<br>☐ No<br>☐ Don't know	
If „Prepared":<br>**Did you touch the raw turkey meat with your fingers?**<br>(WITH PICTURE)	☐ Yes	☐ No	☐ Don't know	
If „Ate":<br>**Was the turkey meat that you ate…**	☐ raw	☐ partially raw (not completely cooked)	☐ completely cooked	☐ Don't know
If „Prepared" or „Ate":<br>**Before preparation, was the turkey meat…**<br>*(WITH PICTURES)*	☐ fresh, e.g. from the butcher or from the meat counter	☐ packaged, e.g. from the refrigerated shelf	☐ frozen (frozen or self-frozen turkey meat)	☐ Don't know

4.2.3

**Duck** e.g. duck breast, leg, wings, whole duck	Prepared<br>☐ Yes<br>☐ No<br>☐ Don’t know		Ate<br>☐ Yes<br>☐ No<br>☐ Don’t know	
If „Prepared“:<br>**Did you touch the raw duck meat with your fingers?**	☐ Yes	☐ No	☐ Don’t know	
If „Ate“:<br>**Was the duck meat that you ate…**	☐ raw	☐ partially raw (not completely cooked)	☐ completely cooked	☐ Don’t know
If „Prepared“ or „Ate“:<br>**Before preparation, was the duck meat…**<br>*(WITH PICTURES)*	☐ fresh, e.g. from the butcher or from the meat counter	☐ packaged, e.g. from the refrigerated shelf	☐ frozen (frozen or self-frozen duck meat)	☐ Don’t know

4.2.4

**Goose** e.g. goose breast, leg, wings, whole goose	Prepared<br>☐ Yes<br>☐ No<br>☐ Don’t know		Ate<br>☐ Yes<br>☐ No<br>☐ Don’t know	
If „Prepared“:<br>**Did you touch the raw goose meat with your fingers?**	☐ Yes	☐ No	☐ Don’t know	
If „Ate“:<br>**Was the goose meat that you ate…**	☐ raw	☐ partially raw (not completely cooked)	☐ completely cooked	☐ Don’t know
If „Prepared“ or „Ate“:<br>**Before preparation, was the goose meat…**<br>*(WITH PICTURES)*	☐ fresh, e.g. from the butcher or from the meat counter	☐ packaged, e.g. from the refrigerated shelf	☐ frozen (frozen or self-frozen goose meat)	☐ Don’t know

4.2.5

**Pork** e.g. roast pork, schnitzel, chop, strips of pork	Prepared<br>☐ Yes<br>☐ No<br>☐ Don’t know		Ate<br>☐ Yes<br>☐ No<br>☐ Don’t know	
If „Prepared“:<br>**Did you touch the raw pork with your fingers?**<br>(WITH PICTURE)	☐ Yes	☐ No	☐ Don’t know	
If „Ate“:<br>**Was the pork that you ate…**	☐ raw	☐ partially raw (not completely cooked)	☐ completely cooked	☐ Don’t know
If „Prepared“ or „Ate“:<br>**Before preparation, was the pork…**<br>*(WITH PICTURES)*	☐ fresh, e.g. from the butcher or from the meat counter	☐ packaged, e.g. from the refrigerated shelf	☐ frozen (frozen or self-frozen pork)	☐ Don’t know

4.2.6

**Beef or veal** e.g. roast beef, veal schnitzel, strips of meat	Prepared<br>☐ Yes<br>☐ No<br>☐ Don’t know		Ate<br>☐ Yes<br>☐ No<br>☐ Don’t know	
If „Prepared“:<br>**Did you touch the raw beef/veal with your fingers?**	☐ Yes	☐ No	☐ Don’t know	
If „Ate“:<br>**Was the beef/veal that you ate…**	☐ raw	☐ partially raw (not completely cooked)	☐ completely cooked	☐ Don’t know
If „Prepared“ or „Ate“:<br>**Before preparation, was the beef/veal…**<br>*(WITH PICTURES)*	☐ fresh, e.g. from the butcher or from the meat counter	☐ packaged, e.g. from the refrigerated shelf	☐ frozen (frozen or self-frozen beef/veal)	☐ Don’t know

4.2.7					
	**Lamb, mutton or sheep meat**, e.g. lamb chop, roast lamb, leg of mutton	Prepared<br>☐ Yes<br>☐ No<br>☐ Don't know		Ate<br>☐ Yes<br>☐ No<br>☐ Don't know	
	If „Prepared":<br>**Did you touch the raw lamb/mutton/sheep meat with your fingers?**	☐ Yes	☐ No	☐ Don't know	
	If „Ate":<br>**Was the lamb/mutton/sheep meat that you ate…**	☐ raw	☐ partially raw (not completely cooked)	☐ completely cooked	☐ Don't know
	If „Prepared" or „Ate":<br>**Before preparation, was the lamb/mutton/sheep meat…**<br>*(WITH PICTURES)*	☐ fresh, e.g. from the butcher or from the meat counter	☐ packaged, e.g. from the refrigerated shelf	☐ frozen (frozen or self-frozen lamb/mutton /sheep meat)	☐ Don't know

**5**	**The following questions are primarily about the dishes you ate or prepared <u>on the 2018/2019 holidays (Christmas, New Year's Eve, New Year)</u>.**<br><br>**For our study, it is important that all study participants answer these questions, regardless of when they were ill.**
**5.1**	**Did you have a <u>fondue meal</u> on one or more of the following days?** Multiple answers are possible. *(WITH PICTURE)*

On 24, 25, or 26 December 2018 (Christmas)	☐ Yes	☐ No	☐ Don't know
On 31 Dezember 2018 (New Year's Eve) or on 1 Januar 2019 (New Year)	☐ Yes	☐ No	☐ Don't know
On another day, but in the 7 days before onset of my illness	☐ Yes	☐ No	☐ Don't know

If „No" or „Don't know", continue with Q5.2.

5.1.1

Wenn „Yes" on Q5.1:
What kind of fondue meal was it? Multiple answers are possible.

Meat fondue	☐ Yes	☐ No	☐ Don't know
Cheese fondue (without meat)	☐ Yes	☐ No	☐ Don't know
Another kind of fondue (without meat)	☐ Yes	☐ No	☐ Don't know

If „Yes" for Cheese fondue or another kind of fondue without meat:
When exactly did you have the fondue meal (without meat)?
Multiple answers are possible if you had fondue on more than one day.

☐ 24 December 2018 (Christmas Eve)
☐ 25 December 2018 (First Christmas holiday)
☐ 26 December 2018 (Second Christmas holiday)
☐ 31 December 2018 (New Year's Eve)
☐ 1 January 2019 (New Year)
☐ On another day, but in the 7 days before onset of my illness
☐ I do not remember.

If „Yes" for Meat fondue:
When exactly did you have the meat fondue meal?
Multiple answers are possible if you had fondue on more than one day.

☐ 24 December 2018 (Christmas Eve)
☐ 25 December 2018 (First Christmas holiday)
☐ 26 December 2018 (Second Christmas holiday)
☐ 31 December 2018 (New Year's Eve)
☐ 1 January 2019 (New Year)
☐ On another day, but in the 7 days before onset of my illness
☐ I do not remember.

**5.1.2**	*If „Yes" for Meat fondue:*<br><br>**What types of meat were offered at the meat fondue meal?**<br>Multiple answers are possible if several types of meats were offered.

Chicken meat	☐ Yes	☐ No	☐ Don't know
If Yes:<br>Did you eat the chicken meat at the meat fondue meal?	☐ Yes	☐ No	☐ Don't know
Turkey meat	☐ Yes	☐ No	☐ Don't know
If Yes:<br>Did you eat the turkey meat at the meat fondue meal?	☐ Yes	☐ No	☐ Don't know
Pork	☐ Yes	☐ No	☐ Don't know
If Yes:<br>Did you eat the pork at the meat fondue meal?	☐ Yes	☐ No	☐ Don't know
Beef or veal	☐ Yes	☐ No	☐ Don't know
If Yes:<br>Did you eat the beef/veal at the meat fondue meal?	☐ Yes	☐ No	☐ Don't know

**5.1.3**	*If „Yes" for Meat fondue:*<br><br>**How was the meat heated for the fondue?**<br>Multiple answers are possible if you had different types of meat fondue meals.

In broth (e.g., fondue chinoise)	☐
In oil (e.g., fondue bourguignonne)	☐
In wine or a mix of broth and wine (e.g., "winemaker's" fondue)	☐
I do not remember.	☐

**5.1.4**	*If „Yes" for Meat fondue:*<br><br>**At the meat fondue meal, did you touch the raw meat with your fingers?**<br>(WITH PICTURE)<br><br>☐ Yes ☐ No ☐ Don't know
**5.1.5**	*If „Yes" for Meat fondue:*<br><br>**At the meat fondue meal, were the raw meat and the heated meat placed on the same plate?**<br><br>☐ Yes ☐ No ☐ Don't know
**5.1.6**	*If „Yes" for Meat fondue:*<br><br>**At the meat fondue meal, were food items offered that are consumed without heating, e.g., salad, raw vegetables, fruit, homemade sauces/dips?**<br><br>☐ Yes ☐ No ☐ Don't know

**5.1.7**	*If „Yes" for Meat fondue:*<br><br>**How many people were present at the meat fondue meal?** Please take yourself into account.<br><br>└─┴─┘ Number of persons ☐ I do not remember.
**5.1.8**	*If „Yes" for Meat fondue:*<br><br>**How many of the people who were present at the meat fondue meal became ill with diarrhea, abdominal pain or vomiting in the following 7 days?**<br>Please take yourself into account if you became ill in the 7 days after the fondue meal.<br><br>└─┴─┘ Number of persons who became ill<br><br>☐ Nobody became ill after the meat fondue meal.<br><br>☐ I do not know.
**5.1.9**	*If „Yes" for Meat fondue:*<br><br>**Where did the meat fondue meal take place?**<br><br>☐ In a private household (at home or at the home of friends/relatives)<br><br>☐ In a restaurant<br><br>☐ Neither in a private household nor in a restaurant<br><br>☐ I do not know.
**5.1.10**	*If „Yes" for Meat fondue:*<br><br>**In which federal state did the meat fondue meal take place?** (PULL-DOWN MENU)
	☐ Baden Wuerttemberg<br>☐ Bavaria<br>☐ Berlin<br>☐ Brandenburg<br>☐ Bremen<br>☐ Hamburg<br>☐ Hesse<br>☐ Mecklenburg – Western Pomerania<br>☐ Lower Saxony<br>☐ North Rhine - Westphalia<br>☐ Rhineland-Palatinate<br>☐ Saarland<br>☐ Saxony<br>☐ Saxony-Anhalt<br>☐ Schleswig-Holsten<br>☐ Thuringia<br>☐ I had meat fondue meals in more than one federal state.<br>☐ Don't know

**5.2**	**Did you have a raclette grill meal (food is prepared in small pans that are heated in a raclette table oven) on one of the following days?** (WITH PICTURE)

On 24, 25, or 26 December 2018 (Christmas)	☐ Yes	☐ No	☐ Don't know
On 31 December 2018 (New Year's Eve) or on 1 January 2019 (New Year)	☐ Yes	☐ No	☐ Don't know
On another day, but in the 7 days before onset of my illness	☐ Yes	☐ No	☐ Don't know

If „No" or „Don't know" continue with Q5.3.

5.2.1

If „Yes" for Q5.2:

When exactly did you have the raclette grill meal?

Multiple answers are possible if you had raclette grill meals on more than one day.

☐ 24 December 2018 (Christmas Eve)
☐ 25 December 2018 (First Christmas holiday)
☐ 26 December 2018 (Second Christmas holiday)
☐ 31 December 2018 (New Year's Eve)
☐ 1 January 2019 (New Year)
☐ On another day, but in the 7 days before onset of my illness
☐ I do not remember.

5.2.2

Was meat offered at the raclette grill meal (as raw meat, e.g., in meat cubes or strips)?

☐ Yes ☐ No ☐ Don't know

5.2.3

If „Yes"to Q5.2.2:

Which types of meat were offered at the raclette grill meal (as raw meat, e.g. in meat cubes or strips)?

Multiple answers are possible if several types of meats were offered.

Chicken meat	☐ Yes	☐ No	☐ Don't know
If Yes: Did you eat the chicken meat at the raclette grill meal?	☐ Yes	☐ No	☐ Don't know
Turkey meat	☐ Yes	☐ No	☐ Don't know
If Yes: Did you eat the turkey meat at the raclette grill meal?	☐ Yes	☐ No	☐ Don't know
Pork	☐ Yes	☐ No	☐ Don't know
If Yes: Did you eat the pork at the raclette grill meal?	☐ Yes	☐ No	☐ Don't know
Beef or veal	☐ Yes	☐ No	☐ Don't know
If Yes: Did you eat the beef/veal at the raclette grill meal?	☐ Yes	☐ No	☐ Don't know

**5.2.4**	*If at least one type of meat was offered at the raclette grill meal:*<br><br>**At the raclette grill meal, did you touch the raw meat with your fingers?**<br><br>☐ Yes ☐ No ☐ Don't know
**5.2.5**	*If at least one type of meat was offered at the raclette grill meal:*<br><br>**At the raclette grill meal, were the raw meat and the heated meat placed on the same plate?**<br><br>☐ Yes ☐ No ☐ Don't know
**5.2.6**	*If at least one type of meat was offered at the raclette grill meal:*<br><br>**At the raclette grill meal, were food items offered that are consumed without heating, e.g., salad, raw vegetables, fruit, homemade sauces/dips?**<br><br>☐ Yes ☐ No ☐ Don't know
**5.2.7**	*If at least one type of meat was offered at the raclette grill meal:*<br><br>**How many people were present at the raclette grill meal?** Please take yourself into account.<br><br>└┴┘ Number of persons ☐ I do not remember.
**5.2.8**	*If at least one type of meat was offered at the raclette grill meal:*<br><br>**How many of the people who were present at the raclette grill meal became ill with diarrhea, abdominal pain or vomiting in the following 7 days?** Please take yourself into account if you became ill in the 7 days after the raclette grill meal.<br><br>└┴┘ Number of persons who became ill<br><br>☐ Nobody became ill after the raclette grill meal.<br><br>☐ I do not know.
**5.2.9**	*If Yes to Q5.2:*<br><br>**Where did the raclette grill meal take place?**<br><br>☐ In a private household (at home or at the home of friends/relatives)<br><br>☐ In a restaurant<br><br>☐ Neither in a private household nor in a restaurant<br><br>☐ I do not remember.

<table>
<tr><td rowspan="2">5.2.10</td><td colspan="2">If Yes to Q 5.2

In which federal state did the raclette grill meal take place? (PULL-DOWN MENU)</td></tr>
<tr><td>☐ Baden Wuerttemberg
☐ Bavaria
☐ Berlin
☐ Brandenburg
☐ Bremen
☐ Hamburg
☐ Hesse
☐ Mecklenburg – Western Pomerania</td><td>☐ Lower Saxony
☐ North Rhine - Westphalia
☐ Rhineland-Palatinate
☐ Saarland
☐ Saxony
☐ Saxony-Anhalt
☐ Schleswig-Holsten
☐ Thuringia
☐ I had raclette grill meals in more than one federal state.
☐ Don’t know</td></tr>
</table>

**5.3**	**One one or more of the following days, did you have a meal where meat was directly prepared at the table (other than fondue or raclette meals), e.g., table grill meals; "Korean barbecue"; hot stone meals)?** (WITH PICTURE)

On 24, 25, or 26 December 2018 (Christmas)	☐ Yes	☐ No	☐ Don't know
On 31 December 2018 (New Year's Eve) or on 1 January 2019 (New Year)	☐ Yes	☐ No	☐ Don't know
On another day, but in the 7 days before onset of my illness	☐ Yes	☐ No	☐ Don't know

If „No" or „Don't know" continue with Q5.4.

5.3.1

If „Yes" to Q5.3:

When exactly did you have a meal where the meat was prepared at the table, e.g., a table grill meal?

Multiple answers are possible if you had more than one of this type of meal.

☐ 24 December 2018 (Christmas Eve)
☐ 25 December 2018 (First Christmas holiday)
☐ 26 December 2018 (Second Christmas holiday)
☐ 31 December 2018 (New Year's Eve)
☐ 1 January 2019 (New Year)
☐ On another day, but in the 7 days before onset of my illness
☐ I do not remember.

5.3.2

If „Yes" to Q5.3:
Which types of meat were offered at this meal (as raw meat, e.g. in meat cubes or strips)?

Chicken meat	☐ Yes	☐ No	☐ Don't know
If Yes: Did you eat the chicken meat at this meal?	☐ Yes	☐ No	☐ Don't know
Turkey meat	☐ Yes	☐ No	☐ Don't know
If Yes: Did you eat the turkey meat at this meal?	☐ Yes	☐ No	☐ Don't know
Pork	☐ Yes	☐ No	☐ Don't know
If Yes: Did you eat the pork at this meal?	☐ Yes	☐ No	☐ Don't know
Beef or veal	☐ Yes	☐ No	☐ Don't know
If Yes: Did you eat the beef/veal at this meal?	☐ Yes	☐ No	☐ Don't know

**5.3.3**	*If „Yes“ to Q5.3:*<br><br>**Did you touch the raw meat with your fingers at this meal?**<br><br>☐ Yes ☐ No ☐ Don’t know
**5.3.4**	*If „Yes“ to Q5.3:*<br><br>**Were the raw meat and the heated meat placed on the same plate at this meal?**<br><br>☐ Yes ☐ No ☐ Don’t know
**5.3.5**	*If „Yes“ to Q5.3:*<br><br>**At this meal, were food items offered that are consumed without heating, e.g., salad, raw vegetables, fruit, homemade sauces/dips?**<br><br>☐ Yes ☐ No ☐ Don’t know
**5.3.6**	*If „Yes“ to Q5.3:*<br><br>**How many people were present at the meal where the meat was prepared directly at the table?** Please take yourself into account.<br><br>└┴┘ Number of persons ☐ I do not remember.
**5.3.7**	*If „Yes“ to Q5.3:*<br><br>**How many of the people who were present at this meal became ill with diarrhea, abdominal pain or vomiting in the following 7 days?** Please take yourself into account if you became ill in the 7 days after this meal.<br><br>└┴┘ Number of persons who became ill<br><br>☐ Nobody became ill after this meal.<br><br>☐ I do not know.
**5.3.8**	*If „Yes“ to Q5.3:*<br>**Where did the meal where the meat was heated directly at the table take place?**<br><br>☐ In a private household (at home or at the home of friends/relatives)<br><br>☐ In a restaurant<br><br>☐ Neither in a private household nor in a restaurant<br><br>☐ I do not remember.

**5.4 (A)**	***If „No" or „Don't know" to Q5.1., 5.2, and 5.3:***<br><br>**On one of the following days, did you have a meal with poultry meat (e.g., chicken, turkey)?** Multiple answers are possible.<br><br>☐ 24 December 2018 (Christmas Eve)<br>☐ 25 December 2018 (First Christmas holiday)<br>☐ 26 December 2018 (Second Christmas holiday)<br>☐ 31 December 2018 (New Year's Eve)<br>☐ 1 January 2019 (New Year)<br>☐ No, on none of these days.<br>☐ I do not remember.<br><br>*If „No" or „Don't remember" continue with Q5.5*
**5.4 (B)**	***If „Yes" to 5.1., 5.2, or 5.3:***<br><br>**<u>In addition</u> to the fondue meal, raclette grill meal or tabletop grill meal, did you have a meal with poultry meat (e.g., chicken, turkey) on one of the following days?** Multiple answers are possible.<br><br>☐ 24 December 2018 (Christmas Eve)<br>☐ 25 December 2018 (First Christmas holiday)<br>☐ 26 December 2018 (Second Christmas holiday)<br>☐ 31 December 2018 (New Year's Eve)<br>☐ 1 January 2019 (New Year)<br>☐ No, on none of these days.<br>☐ I do not remember.<br><br>*If „No" or „Don't remember" continue with Q5.5*

**5.4.1**	*If „Yes“ to Q5.4:*<br><br>**What type of poultry meat was offered at this meal?** Multiple answers are possible.

Chicken Meat	☐ Yes	☐ No	☐ Don't know
If Yes:<br>**Did you eat the chicken meat at this meal?**<br>☐ Yes ☐ No ☐ Don't know			
Turkey meat	☐ Yes	☐ No	☐ Don't know
If Yes:<br>**Did you eat the turkey meat at this meal?**<br>☐ Yes ☐ No ☐ Don't know			
Duck meat	☐ Yes	☐ No	☐ Don't know
If Yes:<br>**Did you eat the duck meat at this meal?**<br>☐ Yes ☐ No ☐ Don't know			
Goose meat	☐ Yes	☐ No	☐ Don't know
If Yes:<br>**Did you eat the goose meat at this meal?**<br>☐ Yes ☐ No ☐ Don't know			
Other poultry meat	☐ Yes	☐ No	☐ Don't know
If Yes:<br>What kind of other poultry meat was this?<br>____			
If Yes:<br>**Did you eat the other poultry meat at this meal?**<br>☐ Yes ☐ No ☐ Don't know			

**5.4.2**	*If „Yes“ to Q5.4:*<br><br>**Did you touch the raw poultry meat with your fingers?**<br>☐ Yes ☐ No ☐ Don't know

5.5	**On which of the following days did you have meals that we have not yet asked you about?** We mean main meals (lunch or dinner). Multiple answers are possible.<br><br>☐ 24 December 2018 (Christmas Eve)<br>☐ 25 December 2018 (First Christmas holiday)<br>☐ 26 December 2018 (Second Christmas holiday)<br>☐ 31 December 2018 (New Year's Eve)<br>☐ 1 January 2019 (New Year)<br>☐ On none of these days.<br>☐ I do not remember.

**5.5.1**	*If 24 December:*<br><br>**Which types of meals/dishes that we have not yet queried did you eat on 24 December?**<br><br>Meal/Dish 1: ______________________<br><br>Meal/Dish 2: ______________________<br><br>Meal/Dish 3: ______________________<br><br>*If 25 December:*<br><br>**Which types of meals/dishes that we have not yet queried did you eat on 25 December?**<br><br>Meal/Dish 1: ______________________<br><br>Meal/Dish 2: ______________________<br><br>Meal/Dish 3: ______________________<br><br>*If 26 December:*<br><br>**Which types of meals/dishes that we have not yet queried did you eat on 26 December?**<br><br>Meal/Dish 1: ______________________<br><br>Meal/Dish 2: ______________________<br><br>Meal/Dish 3: ______________________<br><br>*If 31 December:*<br><br>**Which types of meals/dishes that we have not yet queried did you eat on 31 December?**<br><br>Meal/Dish 1: ______________________<br><br>Meal/Dish 2: ______________________<br><br>Meal/Dish 3: ______________________

	If 1 January:<br><br>**Which types of meals/dishes that we have not yet queried did you eat on 1 January?**<br><br>Meal/Dish 1: ____<br><br>Meal/Dish 2: ____<br><br>Meal/Dish 3: ____

	**The following questions refer to the 7 days before the onset of your illness.**
6	**Raw or unpasteurised milk**
**6.1**	**Raw milk** is **untreated** milk that has not been pasteurized.<br><br>Milk that is offered at retail is **typically pasteurised** (i.e., the milk was heated in a special treatment).<br><br>**Raw milk** can either be obtained directly from the farmer (as **"Milch ab Hof"** [=milk from the farm]), e.g., from automated milk dispensing machines at the farm, or, specially labeled, from certain grocery stores, e.g.,health food stores, as so-called **"Vorzugsmilch"**.<br><br>**In the 7 days before the onset of your illness did you drink raw milk („Milch ab Hof" or „Vorzugsmilch")?**

6.1.1

**"Milch ab Hof "**[WITH PICTURE]	☐ Yes	☐ No	☐ Don't know
If Yes:<br>Was the raw milk („Milch ab Hof") heated before consumption?	☐ Yes, always<br><br>☐ Yes, but not always	☐ No	☐ Don't know

6.1.2

**„Vorzugsmilch"** [WITH PICTURE]	☐ Yes	☐ No	☐ Don't know
If Yes:<br>Was the raw milk („Vorzugsmilch") heated before consumption?	☐ Yes, always<br><br>☐ Yes, but not always	☐ No	☐ Don't know

7	Eating out
7.1	**In the 7 days before the onset of your illness did you eat out, e.g., in a restaurant, canteen, a private household, e.g., at a friend's house?**<br><br>☐ Yes ☐ No ☐ Don't know
7.1.1	*If Yes to Q7.1:*<br>**Where did you eat?** Multiple answers are possible.

In a restaurant/café/bistro	☐ Yes	☐ No	☐ Don't know
At a snack bar/in a fast food restaurant	☐ Yes	☐ No	☐ Don't know
In a canteen	☐ Yes	☐ No	☐ Don't know
In a private household (friends/relatives)	☐ Yes	☐ No	☐ Don't know

7.1.2	*If Yes to Q7.1:*<br><br>**In the 7 days before the onset of your illness did you eat chicken meat when you ate out?**<br><br>☐ Yes ☐ No ☐ Don't know

8	**Information about the ill person**<br>To be able to better analyse the data you provided, we kindly ask you for some additional information about the ill person.
8.1	**To which of the following age groups do you belong?**<br><br>☐ Younger than 80 years<br><br>☐ 80 years or older<br><br>☐ I do not want to answer this question.
8.1.1	*If younger than 80 years:*<br><br>**Please tell us your month and year of birth** [PULLDOWN MENU]**:**<br><br>└─┴─┘ Month of birth └─┴─┴─┴─┘ Year of birth<br><br>☐ I do not want to answer this question.
8.2	**Please specify your gender:**<br><br>☐ Female<br><br>☐ Male<br><br>☐ I do not want to answer this question.
8.3	**What is the postal code of your place of residence?**<br><br>└─┴─┴─┴─┴─┘<br><br>☐ I do not want to answer this question.

This brings you to the end of our survey. Would you like to comment on the survey? Please enter your comments here.

____

____

Thank you very much for taking part in this survey!
Your information is of great value for our study.

Your RKI Team
